# Effect of Na_2_O, MgO, CaO, and Fe_2_O_3_ on Characteristics of Ceramsite Prepared from Lead–Zinc Tailings and Coal Gangue

**DOI:** 10.3390/ma18214928

**Published:** 2025-10-28

**Authors:** Zhongtao Luo, Qi Zhang, Jinyang Guo, Xiaohai Liu, Maoliang Zhang, Xindi Wan, Jiayuan Ye, Lei Liu

**Affiliations:** 1 School of Materials Science and Engineering, Zhengzhou University, Zhengzhou 450001, China; luozhongtao@126.com (Z.L.); zq15836260730@163.com (Q.Z.); 15536873994@163.com (J.G.); liuxiaohai@zzu.edu.cn (X.L.); zhml82@163.com (M.Z.); 2 Henan Building Materials Research and Design Institute Co., Ltd., Zhengzhou 450052, China; wanxindi@163.com; 3 Institute of Cement Science and New Building Materials, China Building Materials Academy, Beijing 100024, China; yejiayuan@cbma.com.cn

**Keywords:** lead–zinc tailings, coal gangue, ceramsite, flux components, microstructure, performance

## Abstract

High-temperature sintering for ceramsite preparation is a safe and effective approach to recycle solid waste. Flux components are critical in ceramsite sintering, as they can reduce sintering temperature, modulate the viscosity and content of the liquid phase, and ultimately optimize ceramsite performance. However, existing studies on lead–zinc tailings (LZTs) and coal gangue (CG)-based ceramsite lack systematic exploration of key fluxes (Na_2_O, MgO, CaO, Fe_2_O_3_), limiting the high-value utilization of these wastes. Under fixed sintering conditions (preheating at 400 °C for 30 min, sintering at 1250 °C for 30 min, heating rate of 10 °C/min), this work systematically investigated the effects of these fluxes (in the forms of carbonates, except for Fe_2_O_3_) on LZTs-CG ceramsite. The mechanical properties, mineral composition, microstructure and heavy metal leaching of samples were analyzed using various methods, including uniaxial compression, X-ray diffraction (XRD), scanning electron microscopy (SEM), and inductively coupled plasma optical emission spectrometry (ICP-OES). Results showed that, while Fe_2_O_3_ exerted a non-monotonic influence, Na_2_O, MgO, and CaO improved apparent density and compressive strength, concurrently reducing water absorption, with these effects enhancing in a dose-dependent manner. Na_2_O, MgO and Fe_2_O_3_ facilitated the formation of labradorite, cordierite and hematite, respectively. All fluxes weakened the diffraction peaks of quartz and mullite. ICP-OES results indicated that the fluxes slightly increased Pb and Zn leaching, yet the highest values (0.1975 mg/L for Pb, 0.0485 mg/L for Zn) were well below the limits specified in the Chinese national standard GB 5086.2-1997 (Leaching Toxicity of Solid Waste—Horizontal Vibration Extraction Procedure). This work shows optimized flux composition enables high-performance, eco-safe LZTs-CG ceramsite, supporting LZTs and CG high-value utilization and sustainable development.

## 1. Introduction

Accelerated industrialization has driven a surge in global mineral resource exploration and production, leading to substantial mining waste issues [1,2]. Lead–zinc tailings (LZTs) are residual solid wastes from lead–zinc ore crushing and flotation [3]. As a major global producer of these ores, China accounts for 47% and 35% of global lead and zinc output, respectively [4,5], generating over 10 million tons of LZTs annually with a utilization rate of only ~7% [6]. Most LZTs are openly stockpiled, occupying vast land and causing gradual heightening of tailings dams via continuous accumulation, which elevates dam safety risks [7,8]. They also contain lead, zinc, and other toxic heavy metals that migrate into surrounding ecosystems, markedly increasing environmental contamination risks [9]. Similarly, coal gangue (CG), a major solid waste from coal mining and washing and one of the most abundant industrial residues in the global coal industry, has a global annual discharge exceeding 1 billion tons, with China alone producing over 300 million tons yearly. This volume is predominantly openly stockpiled, resulting in extremely low utilization efficiency [10,11]. Like LZTs, CG poses prominent environmental and resource challenges, particularly land occupation and pollution [12,13], making the investigation of efficient treatment and recycling approaches for LZTs and CG a prominent research focus in this field.

Ceramsite, an artificial lightweight aggregate with excellent properties (e.g., low bulk density, high compressive strength), is conventionally fabricated from clay, shale, or natural expandable minerals via high-temperature sintering [14,15]. However, as non-renewable resources, clay and shale are being depleted due to over-exploitation, prompting the adoption of industrial solid wastes as alternative raw materials for ceramsite production [16,17,18]. Numerous bulk solid wastes (e.g., fly ash, red mud, iron tailings) have been validated as viable ceramsite feedstocks [19,20,21,22]. Notably, CG is widely employed as a primary raw material for sintered ceramsite, owing to its high silica and alumina content that enhances sintering stability [23]. Similarly, LZTs offer distinct advantages: their alkali metals act as sintering aids to reduce energy consumption, while gaseous products generated during sintering induce a foaming effect favorable for forming a porous ceramsite structure [24]. Thus, building on the aforementioned advantages of CG and LZTs, utilizing LZTs in combination with CG for ceramsite production is highly feasible, thereby facilitating the efficient utilization and resource recycling of these two industrial solid wastes.

Numerous studies confirm that raw material composition and sintering regime are the two core factors governing ceramsite’s sintering quality and performance [17,22,25,26]. Functionally, ceramsite feedstocks comprise three indispensable categories (structural components, gas-generating components, and flux components), with flux components being particularly critical for regulating the sintering process [27]. As validated in previous research, alkali and alkaline earth metal oxides (e.g., Na_2_O, CaO, MgO, and Fe_2_O_3_) act as effective fluxes, reducing sintering temperature and modulating liquid phase viscosity to optimize sintering efficiency [28]. Specifically, for solid waste-based ceramsite, extensive studies have explored the impacts of these oxides as fluxes on sintering performance. For instance, Zou et al. [29] investigated the influences of Fe_2_O_3_, CaO, and MgO on ceramsite synthesized from wastewater and drinking-water treatment sludge, revealing that increasing Fe_2_O_3_ content promoted the formation of complex crystalline phases, reduced pore volume, and thereby enhanced compressive strength. In contrast, higher CaO content increased porosity, while MgO exerted a negligible influence on overall performance. Similarly, Cao et al. [30] prepared ceramsite using municipal solid waste incineration bottom ash sludge (MSWI-BAS) as the main raw material, demonstrating that adjusting the contents of SiO_2_ and CaO improved product performance, whereas excessive Al_2_O_3_ inhibited internal crystallization, resulting in increased porosity and decreased compressive strength. Li et al. [31] evaluated the effects of Na_2_O, MgO, CaO, and Fe_2_O_3_ on the preparation of lightweight aggregate (LWA) and the solidification of chromium (Cr), reporting that Na_2_O significantly promoted both LWA formation and Cr solidification, while CaO, MgO, and Fe_2_O_3_ had only minor regulatory effects.

Notably, these existing studies focus on specific solid waste systems (e.g., sludge, MSWI-BAS), yet lead–zinc tailings (LZTs) and coal gangue (CG)—two of the most abundant industrial solid wastes with substantial resource utilization potential—remain underexplored. Specifically, there is a critical lack of systematic research on how key flux components (Na_2_O, MgO, CaO, Fe_2_O_3_) influence the characteristics of LZTs-CG-based ceramsite. Furthermore, while our previous work [32] has validated the feasibility of preparing high-performance LZTs-CG-based ceramsite (laying the foundation for their large-scale recycling), the underlying mechanisms by which these fluxes regulate sintering behavior, mechanical/physical properties, and heavy metal (Pb, Zn) leaching behavior of the product remain unclear. Given that flux effects are highly dependent on the parent solid waste matrix, this dual knowledge gap not only hinders the rational design of flux compositions for optimizing LZTs-CG ceramsite performance but also limits the practical application of this resource recycling technology. Thus, investigating the roles of Na_2_O, MgO, CaO, and Fe_2_O_3_ in LZTs-CG-based ceramsite is both novel and necessary, as it can fill the existing research void, enable targeted performance optimization, and ultimately facilitate the efficient resource utilization of these two massive industrial solid wastes.

Specifically, this study aims to address the aforementioned knowledge gaps through three core objectives: (i) systematically investigate the effects of Na_2_O, MgO, CaO, and Fe_2_O_3_ on the key properties of LZTs-CG-based ceramsite (including apparent density, compressive strength, and water absorption) under a fixed sintering regime (preheating at 400 °C for 30 min, sintering at 1250 °C for 30 min, heating rate of 10 °C/min); (ii) quantitatively evaluate the leaching behavior of Pb and Zn from the sintered ceramsite to assess its environmental safety; (iii) comprehensively analyze the effects of flux components on the mineral composition and microstructure of ceramsite using scanning electron microscopy (SEM) and X-ray diffraction (XRD). The findings of this study are expected to enrich the theoretical framework for the preparation and performance optimization of LZTs-CG-based ceramsite, while providing targeted technical support for the high-value recycling of LZTs and CG, ultimately contributing to sustainable development in the mining and construction sectors, as well as the alleviation of environmental contamination caused by industrial solid wastes.

## 2. Materials and Methods

### 2.1. Materials

In this study, LZTs, CG, and SiC (serving as the foaming agent) were employed as the raw materials for ceramsite preparation. Specifically, LZTs were sourced from Zhongjin Lingnan Mining Co., Ltd., Nanning, China, while CG was procured from Lixin Coal Mine, Tongling, China. The foaming agent SiC was obtained from a commercial supplier. Furthermore, LZTs and CG were ball-milled for 15 min using an SM-500 ball mill, dried to constant weight at 105 ± 5 °C, and passed through a 0.074 mm sieve. Notably, CG was subjected to additional desulfurization and decarbonization pretreatment before use, aiming to eliminate adverse effects of residual sulfur and carbon on the sintering behavior and mechanical properties of the final ceramsite. The detailed pretreatment procedure for CG was as follows: the sieved CG powder was placed in a muffle furnace, heated from room temperature to 700 °C at a rate of 10 °C/min under air atmosphere, held at 700 °C for 90 min for oxidative decomposition of sulfur-containing and carbonaceous components, and then cooled to room temperature along with the furnace to obtain pretreated CG with significantly reduced sulfur and carbon contents.

The chemical compositions of pretreated LZTs and CG are presented in Table 1, and the corresponding test methods are detailed in the subsequent Section 2.3.1.

The flux components employed in this study included Na_2_O, MgO, CaO, and Fe_2_O_3_. These oxides were introduced via their respective carbonate or oxide precursors to ensure uniform dispersion in the raw material matrix: specifically, Na_2_O was incorporated as sodium carbonate (Na_2_CO_3_), MgO as magnesium carbonate (MgCO_3_), CaO as calcium carbonate (CaCO_3_), and Fe_2_O_3_ as analytical-grade iron oxide powder. All chemical reagents (Na_2_CO_3_, MgCO_3_, CaCO_3_, and Fe_2_O_3_) were of analytical grade and purchased from Sinopharm Chemical Reagent Co., Ltd., Shanghai, China.

**Figure 1:**
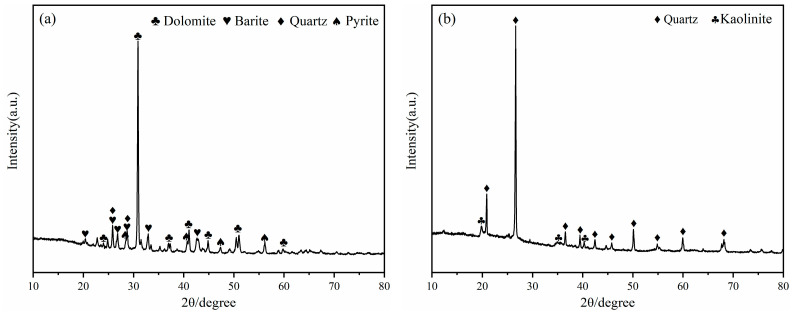
XRD patterns of LZTs (**a**) and CG (**b**).

**Figure 2:**
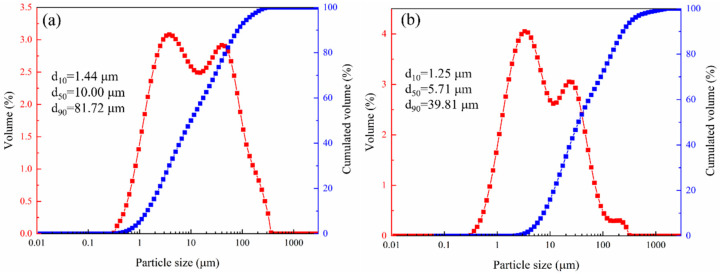
The particle size distribution of LZTs (**a**) and CG (**b**).

The mineral composition of LZTs and CG was analyzed by XRD, and the results are presented in Figure 1. The minerals in LZTs were primarily dolomite (CaMg(CO_3_)_2_, PDF #36-0426), barite (BaSO_4_, PDF #80-0512), quartz (SiO_2_, PDF #46-1045) and pyrite (FeS_2_, PDF #71-2219), while the minerals in CG were mainly quartz (SiO_2_, PDF #46-1045) and Kaolinite (Al_2_Si_2_O_5_(OH)_4_, PDF #78-2109). Meanwhile, the particle size distribution of LZTs and CG were tested using a laser particle size analyzer, as shown in Figure 2. The median particle size of LZTs and CG was 10.00 μm and 5.71 μm, respectively.

### 2.2. Sample Preparation

**Figure 3:**
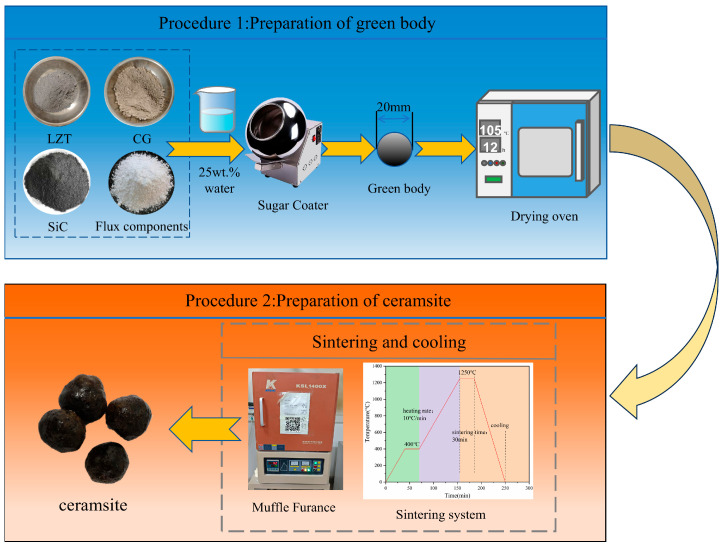
The preparation procedures of the ceramsite in this study.

Several experimental parameters were determined based on our prior research [32]. For instance, an LZTs:CG mass ratio of 2:8 was selected as the matrix ratio for ceramsite preparation, with the same preparation process as reported previously employed. In the present study, the preparation experiments were conducted under a fixed sintering regime: preheating at 400 °C for 30 min, sintering at 1250 °C for 30 min, and a heating rate of 10 °C/min. The remaining detailed experimental procedures are presented in Figure 3.

The mass ratios of the four flux components (Na_2_O, MgO, CaO, Fe_2_O_3_) to the LZTs-CG ceramsite base matrix are presented in Table 2. Notably, in the ceramsite preparation process, the dosages of these flux components were calculated on an oxide basis (hereafter consistently applied for all subsequent flux dosage calculations). All flux components were incorporated as external admixtures—i.e., added separately to the preblended LZTs-CG base matrix.

The specific addition levels of each flux component (by mass of the total base matrix) were as follows: Na_2_O and MgO were each tested at 1%, 2%, and 5%; CaO and Fe_2_O_3_ were tested at 1%, 2%, 5%, and an additional 10%. This differential design for CaO and Fe_2_O_3_ was based on two key considerations [25,29]: (i) CaO acts as a flux that can promote liquid phase formation during sintering—exploring a 10% high addition level helps clarify the threshold at which excessive CaO may induce over-sintering; (ii) Fe_2_O_3_ not only modulates sintering behavior but also influences ceramsite performance—adding a 10% level enables investigation of its dual effects on composition, microstructure and mechanical properties under high-concentration conditions. This expanded testing range ensures comprehensive coverage of both moderate and high flux dosages, which is critical for optimizing flux addition and avoiding performance degradation in LZTs-CG based ceramsite.

### 2.3. Characterization

#### 2.3.1. Chemical Composition Analysis

X-ray fluorescence spectroscopy (XRF, PANalytical B.V., Almelo, The Netherlands) was used to determine the chemical compositions of pretreated LZTs and CG. Calibration was performed using silicate-matched certified reference materials (CRMs, GBW 07405), which cover both main elements (Si, Al, Fe, Ca) and characteristic elements (Pb, Zn, S) of the samples. The calibration curve exhibited a fitting degree (R^2^) ≥ 0.995. Loss on ignition (LOI) was measured to correct the XRF results. Briefly, 2.0000 g of dried sample was placed in a pre-weighed platinum crucible, heated to 550 °C for organic matter removal, then further heated to 950 °C for carbonate and crystal water decomposition. After cooling to room temperature in a desiccator, LOI was calculated from the mass loss.

#### 2.3.2. Apparent Density Test

According to the Chinese national standard GB/T 17431.2-2010 (Lightweight Aggregates and Its Test Methods—Part 2: Test Methods) [33], the apparent density (ρ) of ceramsite was determined by the water flooding method. Its numerical calculation is carried out using Equation (1):

(1) $$\rho =\frac{m}{V_{2}-V_{1}}$$

where m was the weight of ceramsite (g), V_1_ and V_2_ were the volume of water before and after the ceramsite sample was immersed in water, respectively (mL).

#### 2.3.3. Compressive Strength Test

As in previous studies [34,35], the compressive strength of individual ceramsites was evaluated by averaging the individual compressive strengths of five ceramsites in the test. In this study, individual ceramsite specimens were subjected to compressive loading using a computer-controlled compression testing machine (WHY-300/10, Jinan Shijin Group Co., Ltd., Shandong, China) until rupture. The force values at rupture were recorded, and the individual compressive strength was calculated using Equation (2).

(2) $$P=\frac{2.8F_{C}}{\pi d^{2}}$$

where P was the compressive strength of ceramsite single grain (MPa), F_C_ was the fracture load (N), and d was the diameter of ceramsite (mm).

#### 2.3.4. Water Absorption Test

The water absorption of ceramsite was also tested according to the Chinese national standard GB/T 17431.2-2010, and the value was characterized by the average value of the 1 h water absorption of ceramsite in three measurements in the test. The water absorption of the ceramsite was calculated according to Equation (3).

(3) $$W=\frac{m_{2}-m_{1}}{m_{1}}\times 100\%$$

where W was the water absorption of ceramsite (%), m_1_ and m_2_ were the mass of ceramsite in dry state and saturated surface dry state (g), respectively.

#### 2.3.5. Heavy Metals Leaching Test

The leaching test for heavy metals in ceramsite was conducted in accordance with the Chinese national standard GB 5086.2-1997 (Leaching Toxicity of Solid Waste—Horizontal Vibration Extraction Procedure) [36], and the leaching concentrations of target heavy metals were determined using an inductively coupled plasma optical emission spectrometer (ICP-OES, ICPE-9820, Shimadzu Corporation, Kyoto, Japan). Before the test, the leachate was filtered through a 0.45 μm filter membrane, and the detection limit of the instrument was ≤0.01 mg/L. During the test, the ceramsite sample is ground into powder and mixed with deionized water at a ratio of 1:200. After stirring at 3000 rpm for 120 min, the clarified supernatant is filtered and the well solution to be tested is separated.

#### 2.3.6. X-Ray Diffraction (XRD) Analysis

The mineral composition of the ceramsite samples was analyzed using a D8 Advance X-ray diffractometer (XRD, Bruker Optics, Karlsruhe, Germany) with CuKα_1,2_ radiation (λ = 0.15418 nm). The diffractometer was operated at 40 kV (voltage) and 40 mA (current), with a scanning rate of 4°/min, a 2θ scanning range of 10°−80°, and a step size of 0.02°. The obtained XRD patterns were analyzed using Jade Software Version 6.5.

#### 2.3.7. Scanning Electron Microscopy (SEM) Analysis

The ceramsite was crushed into small fragments. The microscopic morphology of the ceramsite samples was observed using a scanning electron microscope (Gemini SEM 300, Carl Zeiss AG, Jena, Germany). Before the test, the sample was subjected to gold spraying treatment (gold spraying thickness 5-10 nm), with an acceleration voltage of 15 kV.

## 3. Results

### 3.1. Apparent Density and Compressive Strength

**Figure 4:**
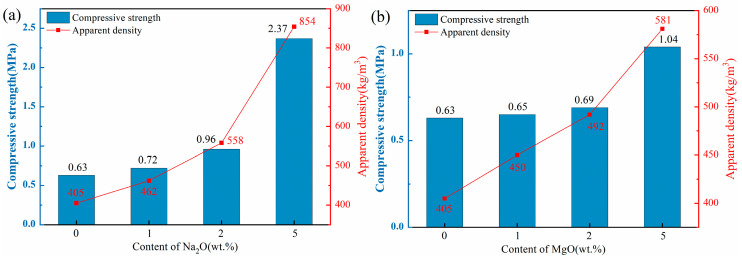

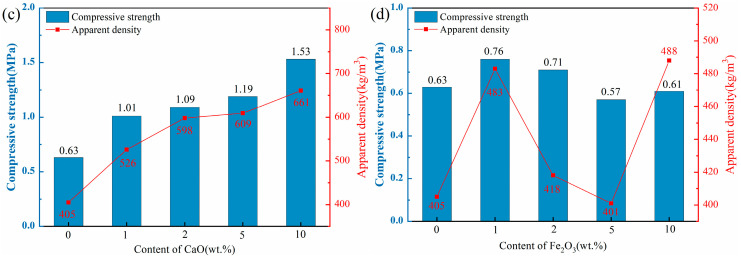
Effect of different flux components on apparent density and compressive strength of ceramsite, (**a**): Na_2_O; (**b**) MgO; (**c**) CaO; (**d**) Fe_2_O_3_.

Figure 4 illustrates the apparent density and compressive strength of ceramsite doped with varying contents of flux components. The apparent densities of all ceramsite groups ranged from 401 to 854 kg/m^3^, below the standard limit of 2000 kg/m^3^. Consequently, each sample group met the basic ceramsite criteria.

As depicted in Figure 4a,b, the apparent density and compressive strength of the ceramsite substantially rose with higher Na_2_O and MgO component levels. Notably, the apparent density and compressive strength of the Na1 sample increased by 14% compared to the control. However, with a Na_2_O content increase to 5 wt.%, these two indexes of the Na5 sample surged by 111% and 276%, respectively, in comparison with the control. The observed enhancement trends could be attributed to the addition of alkali flux Na_2_O that lowered the sintering temperature and widened the sintering temperature range, thus providing a substantial quantity of high-temperature liquid phase. This significantly reduced the number of pores [37]. Furthermore, the incorporation of the CaO component, as depicted in Figure 4c, demonstrated a consistent trend with the aforementioned Na_2_O and MgO. The apparent density and compressive strength of the samples escalated in conjunction with the increment in CaO content. Notably, the Ca10 sample exhibited a remarkable enhancement of 63% and 143% in apparent density and compressive strength, respectively, when compared to the control. The incorporation of CaO could facilitate the formation of the crystalline phase by the excess Ca with the Si-Al system, thereby enhancing the structural integrity of the ceramsite [38,39], which was confirmed by the subsequent XRD and SEM analyses.

In contrast to other flux components, the influence of Fe_2_O_3_ on ceramsite properties was more complex. As shown in Figure 4d, with increasing Fe_2_O_3_ content, the compressive strength of ceramsite exhibited a non-monotonic trend: initial increase, subsequent decrease, and final recovery. Specifically, at 1 wt.% Fe_2_O_3_, the apparent density and compressive strength of the samples were higher than those of the control group. When Fe_2_O_3_ content increased to 5 wt.%, both indexes decreased. Further increasing Fe_2_O_3_ to 10 wt.% led to the recovery of apparent density and compressive strength. This complex variation can be closely associated with the dual role of Fe_2_O_3_ in the sintering process: (i) Redox-induced pore evolution: At high sintering temperatures, Fe^3+^ in Fe_2_O_3_ underwent stepwise reduction (Fe_2_O_3_→FeO), releasing trace oxygen. At 1 wt.% Fe_2_O_3_, the released oxygen generated small, uniformly distributed pores, while the moderate liquid phase (promoted by Fe_2_O_3_ as a flux) enhanced structural compactness—collectively improving strength and density. At 5 wt.% Fe_2_O_3_, excessive oxygen release leaded to pore coarsening and uneven distribution, and the liquid phase was insufficient to fill these large pores, resulting in decreased strength and density. (ii) Liquid phase regulation: When Fe_2_O_3_ content reached 10 wt.%, its strong fluxing effect significantly promoted a substantial amount of liquid phase to fill partial pores and densify the structure—thus reversing the downward trend of apparent density and compressive strength. This mechanism was consistent with the findings of [40,41], which confirmed that high Fe_2_O_3_ content could mitigate pore-induced performance degradation by optimizing liquid phase behavior.

### 3.2. Water Absorption

**Figure 5:**
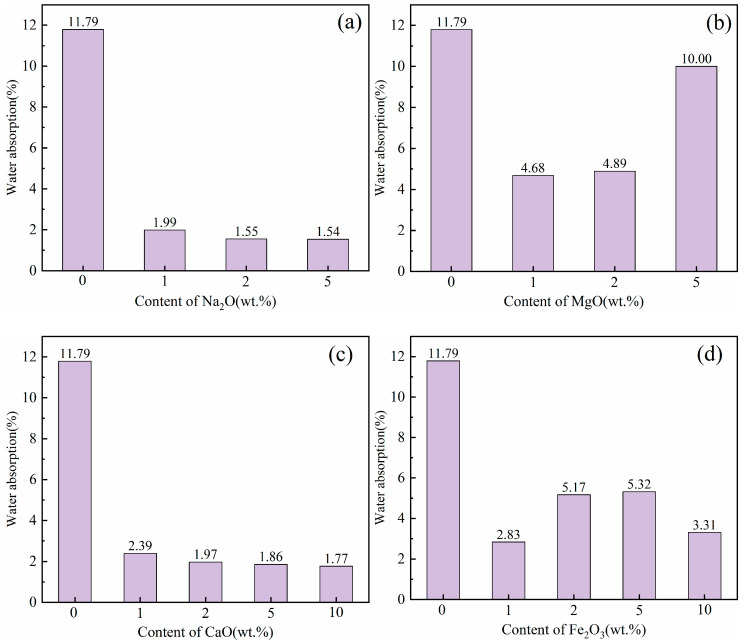
Effect of different flux components on water absorption of ceramsite, (**a**): Na_2_O; (**b**) MgO; (**c**) CaO; (**d**) Fe_2_O_3_.

Figure 5 illustrates the water absorption of ceramsite mixed with varying flux component contents. The water absorption of the control group was 11.79%, while all experimental groups exhibited a reduction (the maximum decrease was 87%), indicating that the flux components exert a more pronounced influence on the water absorption of ceramsite.

The introduction of the Na_2_O component significantly reduced the water absorption of the ceramsite. With the addition of 1 wt.% Na_2_O, water absorption decreased from 11.79% (control) to 1.99%. When the Na_2_O addition further increased to 5 wt.%, water absorption was further reduced to 1.54%. The observed decline in water absorption was mainly attributed to the addition of Na_2_O, which leaded to an increase in the amount of liquid phase, thereby enhancing the densification of the ceramsite matrix [42]. The addition of the MgO component similarly reduced the water absorption of ceramsite. However, the highest water absorption of 10.00% was achieved when the MgO content reached 5 wt.%, although it was still lower than that of the control. This occurrence deviated from the typical trend of reduced water absorption as apparent density and compressive strength increased. This anomaly could be attributed to the heterogeneity in raw material mixing, thereby causing an abnormal increase in water absorption. The incorporation of the CaO component consistently reduced the water absorption of the ceramsite, decreasing from 11.79% (control) to 1.77% as the CaO content continuously increased to 10 wt.%. Notably, this reduction in water absorption shows a positive correlation with increasing CaO content. Specifically, the incorporation of CaO facilitates the formation of more dense crystalline phases, thereby enhancing the structural integrity of the ceramsite (see the following analysis of mineral composition and microstructure). The influence of the Fe_2_O_3_ component on the water absorption of ceramsite was complex. The water absorption for the sample of 1 wt.% Fe_2_O_3_ was 2.83%, increasing to 5.17% and 5.32% as the Fe_2_O_3_ content was raised to 2 wt.% and 5 wt.%, respectively, yet both values remained below that of the control. Then, water absorption decreased again to 3.31% when Fe_2_O_3_ addition was further increased to 10 wt.%. The incorporation of Fe_2_O_3_ might have resulted in a reduction in the number of open pores on the surface and a shift in the ratio of open to closed pores, which consequently led to a decrease in water absorption.

### 3.3. Heavy Metals Leaching Test

Table 3 presents the leaching concentrations of Pb and Zn from the LZTs-CG based ceramsite samples with different flux component additions. All leaching tests were conducted in accordance with the Chinese national standard GB 5086.2-1997, which specifies the maximum allowable leachate concentrations of 5 mg/L for Pb and 100 mg/L for Zn.

Compared to the control sample, the ceramsite samples with flux additions exhibited slightly elevated Pb and Zn leaching concentrations. However, all detected values remained at extremely low levels. Specifically, the highest Pb leaching concentration (0.1975 mg/L) was observed in the Mg5 sample (5 wt.% MgO addition), while the highest Zn leaching concentration (0.0485 mg/L) was detected in the Ca10 sample (10 wt.% CaO addition).

Notably, all measured Pb and Zn leaching concentrations were far below the respective limits specified in GB 5086.2-1997 (Pb: 5 mg/L; Zn: 100 mg/L). This result confirms that the flux-modified LZTs-CG based ceramsite complies with environmental safety standards, verifying its feasibility for practical engineering application from the perspective of heavy metal pollution risk.

### 3.4. XRD Analysis

**Figure 6:**
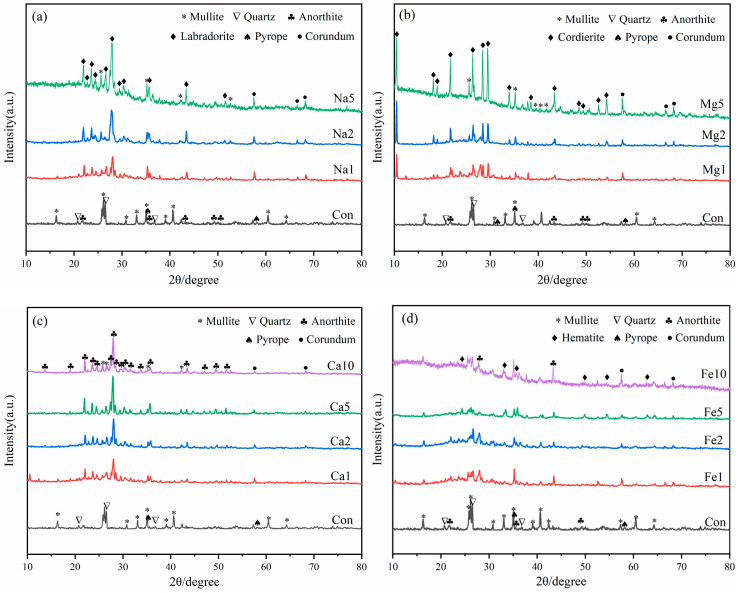
XRD patterns of ceramsite samples with different flux components, (**a**): Na_2_O; (**b**) MgO; (**c**) CaO; (**d**) Fe_2_O_3_.

The effect of different flux component additions on the mineral composition of LZTs-CG based ceramsite was investigated using XRD, with the results presented in Figure 6. Notably, no Pb- or Zn-bearing minerals were detected in any sample. which indicates that Pb and Zn ions existed as solid solution ions in other aluminosilicate phases or in molten amorphous phases during ceramsite sintering, and this is also the primary reason for the extremely low leaching concentrations of these heavy metals (see Section 3.3).

First, as observed in Figure 6, the sintered control sample contained newly formed minerals including mullite (Al_6_Si_2_O_13_, PDF #79-1275), anorthite (CaAl_2_Si_2_O_8_, PDF #89-1459), and pyrope (Mg_3_Al_2_Si_3_O_12_, PDF #73-2366), along with residual quartz. Notably, the dolomite, barite, pyrite, and kaolinite minerals presented in the original LZTs and CG raw materials disappeared, indicating that these minerals have undergone transformation reactions during sintering. Furthermore, it is noteworthy that diffraction peaks of corundum (Al_2_O_3_, PDF #74-1081) were detected in all flux-added ceramsite samples. This is presumably attributed to the fact that flux addition reduced the decomposition temperature of mullite to varying degrees. Specifically, the Si sources released from mullite decomposition were preferentially consumed in other reactions, leading to the enrichment of Al sources and subsequent formation of corundum [43,44].

As shown in Figure 6a, With the incorporation of Na_2_O, a new mineral labradorite ((Ca, Na)(Si, Al)_4_O_8_, PDF #78-0433) formed and its diffraction peak intensity increased with increasing Na_2_O dosage. This is presumably because the addition of Na_2_O enabled Na^+^ ions to incorporate into the aluminosilicate framework alongside the pre-existing Ca^2+^ ions, thereby forming labradorite with a mixed Ca-Na composition. Additionally, the diffraction intensities of mullite and quartz obviously decreased with higher Na_2_O addition, accompanied by the growing prominence of diffuse peaks in the 20–35° range. This phenomenon is attributed to the prominent fluxing effect of alkali flux Na_2_O, which specifically lowered the eutectic temperature of the LZTs-CG system, thereby facilitating the dissolution and melting of crystalline mullite and quartz [42,45].

For samples with MgO addition (Figure 6b), the diffraction peaks of mullite and quartz weakened as MgO content increased. Simultaneously, a new mineral cordierite (Mg_2_Al_4_Si_5_O_18_, PDF #84-1219) emerged, and its diffraction peak intensity increased with increasing MgO dosage. This cordierite formation was attributed to the increased Mg^2+^ concentration, which promoted the reaction between mullite, quartz and Mg^2+^ ions during high-temperature sintering, thus facilitating the synthesis of Mg-rich cordierite [46,47]. Additionally, diffuse peaks also gradually became prominent in the 20–35° range with increasing MgO dosage, indicating that the incorporation of MgO also facilitated the transformation of crystalline phases into the molten phase to a certain extent.

In the CaO-added samples (Figure 6c), the main minerals remained consistent with the control group (mullite, quartz, anorthite, and pyrope), but their peak intensities varied with CaO dosage, with anorthite diffraction peaks strengthening and those of mullite and quartz weakening. Pyrope diffraction peaks remained essentially unchanged. This suggests that most of the added CaO presumably participated in the formation of anorthite by promoting the reaction between mullite, quartz and Ca^2+^ [48]. This phase evolution was consistent with the reduced water absorption of CaO-modified samples (Section 3.2), as the enhanced anorthite formation (a dense crystalline phase) reduces the number of open pores on the ceramsite surface [39].

For samples with Fe_2_O_3_ addition (Figure 6d), diffraction peaks of hematite (Fe_2_O_3_, PDF #85-0987) were observed, with their intensities increasing as Fe_2_O_3_ dosage increased. This indicates that a fraction of the added Fe_2_O_3_ remained unreacted during high-temperature sintering. The other fraction of Fe_2_O_3_ presumably exerted fluxing effects that promoted the melting of crystalline phases, which could be confirmed by the emergence of diffuse peaks in the 20–35° range.

### 3.5. SEM Analysis

**Figure 7:**
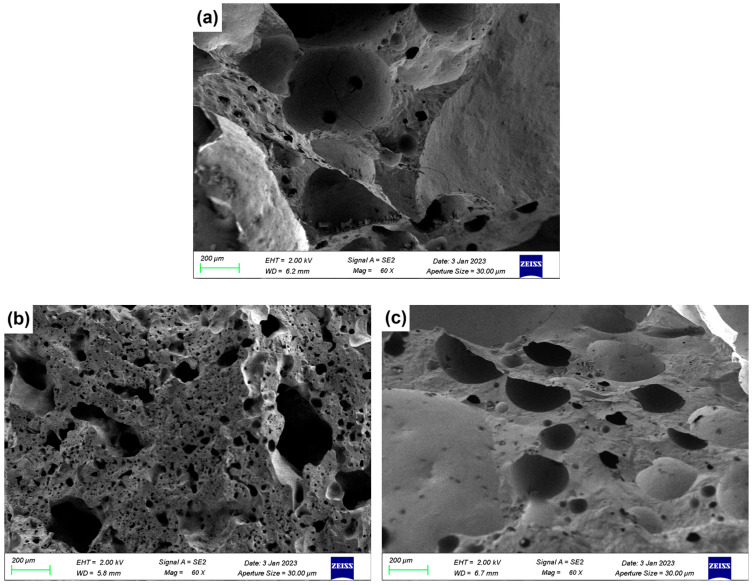

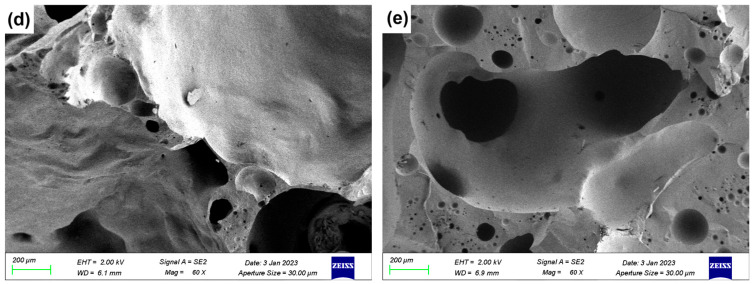
SEM images of ceramsite with different flux components added: (**a**) Con; (**b**) Na5; (**c**) Mg5; (**d**) Ca5; (**e**) Fe5.

Figure 7 presents the SEM micrographs of LZTs-CG based ceramsite samples with different flux component additions, revealing the evolution of pore structure and matrix densification.

As shown in Figure 7a (control sample, no flux addition), the cross-section exhibited numerous irregularly shaped pores with non-uniform size distribution and incomplete pore wall densification. This porous was attributed to the limited liquid phase formation in the unmodified LZTs-CG system, which failed to effectively coat and bond solid particles, which was consistent with the control sample’s relatively low compressive strength (Section 3.1).

Figure 7b shows the micrograph of the 5 wt.% Na_2_O-added sample (Na5 sample). Compared to the control, the Na5 sample exhibited distinct melting behavior: the solid particles were effectively bonded together, the number of macropores was significantly reduced and replaced by the formation of micropores, resulting in a remarkable improvement in the overall densification of the ceramsite matrix. This phenomenon could be explained by the fluxing effect of Na_2_O: as a strong alkali flux, Na_2_O lowered the sintering temperature of the LZTs-CG system and facilitated the generation of a substantial amount of liquid phase. As observed in the 5 wt.% MgO-added sample (Mg5) and presented in Figure 7c, the pore size decreased compared to the control sample, and adhesion of molten substances was observed on its fracture surface. This phenomenon is associated with MgO’s dual role: on one hand, it promoted cordierite formation (confirmed by XRD in Figure 6b), enhancing matrix bonding. On the other hand, its incorporation induced liquid phase formation, further strengthening bonding and thus improving ceramsite matrix densification.

For the CaO-added samples, Figure 7d (5 wt.% CaO-added sample, Ca5) exhibits significant integrity and smoothness of the fracture surface with a more stable ceramsite matrix compared to the control sample. This observation was consistent with the XRD result that most CaO converts to anorthite (CaAl_2_Si_2_O_8_): the formation of this dense crystalline phase strengthened the matrix directly contributing to the reduced water absorption and increased compressive strength of CaO-modified samples.

In the Fe_2_O_3_-added sample (Figure 7e, 5 wt.% Fe_2_O_3_-added sample, Fe5), there are obvious interconnected pores. This was primarily due to the local reducing atmosphere generated by trace organic matter in LZTs and CG, under which Fe_2_O_3_ underwent redox reactions, increasing gas yield [40,41]. The excess gas aggregated and percolated to form interconnected pores when the liquid phase could not fully encapsulate the gas.

## 4. Conclusions

This study systematically investigated the effects of four flux components (Na_2_O, MgO, CaO, Fe_2_O_3_) on the performance, heavy metal solidification, mineral composition, and microstructure of ceramsite prepared from LZTs and CG. The key findings are as follows:

(1) Performance: Na_2_O, MgO, and CaO enhanced apparent density/compressive strength and reduced water absorption with higher dosage. 5 wt.% Na_2_O (Na5) achieved the most significant improvement—apparent density increased by approximately 111% to 854 kg/m^3^ and compressive strength by approximately 276% to 2.37 MPa compared to the control (405 kg/m^3^, 0.63 MPa). This superiority stems from the melting-liquid phase effect induced by the alkali flux Na_2_O, which optimized the pore structure and strengthened the matrix. Fe_2_O_3_ exhibited a non-monotonic effect (increase → decrease → recovery): 5 wt.% Fe_2_O_3_ caused pore coarsening and interconnection (minimum density 401 kg/m^3^, strength 0.57 MPa), while 10 wt.% reversed degradation via liquid phase densification, providing guidance for sintering dosage control. All fluxes reduced water absorption, with 5 wt.% Na_2_O achieving a maximum reduction of 87% (compared to the control sample, 11.79% water absorption).
(2) Environmental safety: Fluxes slightly increased Pb/Zn leaching (undetected in control), but all values (max Pb 0.1975 mg/L, max Zn 0.0485 mg/L) were far below the Chinese national standard GB 5086.2-1997 limits (Pb 5 mg/L, Zn 100 mg/L). The low leaching was primarily attributed to Pb and Zn ions being solidified into the aluminosilicate matrix (either lattice incorporation or amorphous encapsulation), confirming eco-safety.
(3) Mineral evolution: Control ceramsite was dominated by mullite, quartz, anorthite, and pyrope. Corundum emerged in all flux-added samples, attributed to fluxes promoting mullite decomposition—released silicon sources were preferentially consumed in other reactions, leading to aluminum source enrichment and subsequent corundum formation. Specifically, Na_2_O facilitated labradorite formation, MgO promoted cordierite, and Fe_2_O_3_ induced hematite. All fluxes weakened the diffraction peaks of quartz and mullite.
(4) Microstructure: Flux type significantly modified LZTs-CG ceramsite microstructure. The control sample had irregular, uneven pores and incomplete pore wall densification (limited liquid phase). 5 wt.% alkali flux Na_2_O enhanced densification via abundant liquid phase (melting behavior, macropore reduction). 5 wt.% alkaline earth flux MgO reduced pore size and strengthened bonding (cordierite & liquid phase). 5 wt.% alkaline earth flux CaO yielded a smooth, stable matrix (anorthite formation). 5 wt.% Fe_2_O_3_ induced interconnected pores (redox gas generation).

Collectively, this study demonstrates that rational flux regulation enabled the preparation of high-performance, environmentally safe LZTs-CG ceramsite, providing technical support for the high-value recycling of these two industrial solid wastes.

## Figures and Tables

**Table 1:** Chemical compositions of raw material (wt. %).

	SiO_2_	Al_2_O_3_	CaO	Fe_2_O_3_	Na_2_O	K_2_O	MgO	PbO	ZnO	SO_3_	Others	LOI
LZTs	3.52	1.49	22.20	8.59	0.68	0.23	13.68	0.43	0.55	15.06	11.84	21.73
CG	61.63	27.36	0.31	2.82	0.12	0.07	0.54	-	-	0.08	3.19	3.88

**Table 2:** Formulations of ceramsite (wt.%).

LZTs	CG	SiC	Water	Flux Components	Content	Sample Code
20	80	0.5	25	-	-	Con
				Na_2_O	1	Na1
					2	Na2
					5	Na5
				MgO	1	Mg1
					2	Mg2
					5	Mg5
				CaO	1	Ca1
					2	Ca2
					5	Ca5
					10	Ca10
				Fe_2_O_3_	1	Fe1
					2	Fe2
					5	Fe5
					10	Fe10

**Table 3:** Leaching concentrations of Pb and Zn in ceramsite with different flux component contents.

Sample Code	Pb Leaching Concentration (mg/L)	Zn Leaching Concentration (mg/L)
Con	N	N
Na5	0.1564	0.0387
Mg5	0.1975	0.0016
Ca10	0.1255	0.0485
Fe10	0.1667	0.0302

Note: “N” represents not detected.

## Data Availability

The original contributions presented in this study are included in the article. Further inquiries can be directed to the corresponding authors.
